# Resveratrol significantly improves the fertilisation capacity of bovine sex-sorted semen by inhibiting apoptosis and lipid peroxidation

**DOI:** 10.1038/s41598-018-25687-z

**Published:** 2018-05-15

**Authors:** Chong-Yang Li, Ya-Han Zhao, Hai-Sheng Hao, Hao-Yu Wang, Jin-Ming Huang, Chang-Liang Yan, Wei-Hua Du, Yun-Wei Pang, Pei-Pei Zhang, Yan Liu, Hua-Bin Zhu, Xue-Ming Zhao

**Affiliations:** 1 Embryo Biotechnology and Reproduction Laboratory and the Centre of Domestic Animal Reproduction & Breeding, Institute of Animal Sciences (IAS), Chinese Academy of Agricultural Sciences (CAAS), No. 2 Yuanmingyuan Western Road, Haidian District, Beijing, 100193 P.R. China; 2 Dairy Cattle Research Center, Shandong Academy of Agricultural Sciences, No. 159 North of Industry Road, Jinan, Shandong 250131 P.R. China; 3 Livestock and Poultry Import & Export Dept, China Animal Husbandry Group (CAHG), Building 18, Block 8, 188 West Road, South 4th Ring Road, Beijing, 100070 P.R. China

## Abstract

The aim of this study was to test the effects of five different concentrations (0, 10^−3^, 10^−4^, 10^−5^, and 10^−6^ M) of resveratrol (Res) supplementation in bull sperm washing and fertilisation medium on levels of reactive oxygen species (ROS), phosphatidylserine (PS) externalisation, mitochondrial membrane potential (Δψm), ATP and malondialdehyde (MDA), acrosomal integrity, blastocyst rate, and blastocyst quality after *in vitro* fertilisation (IVF). The results for sex-sorted sperm from three bulls showed: (1) ROS and MDA levels in 10^−3^ M and 10^−4^ M Res groups were significantly lower than those of controls (*P* < 0.05); (2) the percentage of viable sperm, percentage of sperm with high Δψm, and the ATP content in 10^−3^ M and 10^−4^ M Res groups were significantly higher than those of controls (*P* < 0.05); (3) the percentage of viable sperm with acrosomal integrity, and the blastocyst percentage and quality of the 10^−4^ M Res group were significantly higher than those of controls (*P* < 0.05). In conclusion, 10^−4^ M Res supplementation in washing and fertilisation medium of sex-sorted bull sperm significantly decreased ROS, PS externalisation, and MDA, and protected mitochondrial function and acrosomal integrity, thereby increasing blastocyst percentage and quality following IVF.

## Introduction

In the dairy cattle industry, the ability to control the sex of calves presents a significant advantage for maximising profit from cattle breeding and dairy farming. Initially developed by Johnson *et al*.^1^, sperm sex sorting by flow cytometry is now widely used to sort X and Y bearing sperm and control the sex of calves by using artificial insemination. At present, artificial insemination of sex-sorted semen can achieve a female calf rate of >90%^2^. Additionally, more than 40% of embryo transfers across the world use embryos produced *in vitro* by *in vitro* fertilisation (IVF), and sex-sorted sperm plays an important role in the commercial production of sexed embryos by IVF^3,4^.

Both flow cytometry sorting^5^ and cryopreservation^6^ greatly increase reactive oxygen species (ROS) levels in sperm. ROS is the main inducer of lipid peroxidation (LPO) in sperm because their plasma membranes are rich in polyunsaturated fatty acids, the principal target for oxidation^7^, and this decreases sperm motility and viability^8^. ROS also cause DNA fragmentation, altering cellular communication and enzymatic pathways^9^, leading to motility loss, disruption of membrane fusion events, poor fertilisation rate, and impaired embryogenesis^10^. Meanwhile, sperm cytoplasm is mainly restricted to the midpiece, in which very few antioxidant mechanisms operate^7,11^, and antioxidants are further diminished when freezing spermatozoa due to the large dilution during the cryopreservation procedure^5^. Furthermore, freeze-thawed bull spermatozoa are more easily peroxidised than fresh spermatozoa^12^. Thus, lowering ROS levels in sex-sorted sperm remains a priority.

Resveratrol (Res) is a natural grape-derived phytoalexin^13^ possessing stronger antioxidant activity than vitamins E and C, as well as lower toxicity^14^. Res acts both in the initiation and propagation of the oxidative process^14^, and can access peroxidised rigid membranes and increase membrane fluidity^12,15,16^. Researchers have successfully utilized Res to stimulate energetic metabolism in spermatozoa to improve viability and motility^6,16^. However, the effects of Res on the quality of sex-sorted bull sperm remain poorly understood, as do the mechanisms involved.

In the present study, five different concentrations (0, 10^−3^, 10^−4^, 10^−5^, and 10^−6^ M) of Res were supplemented in sex-sorted bull sperm washing and fertilisation medium. After washing twice with washing medium and incubating in fertilisation medium for 1.5 h, sex-sorted sperm from three bulls (005x, 011x, and 056x) were tested for ROS, phosphatidylserine (PS) externalisation, mitochondrial membrane potential (Δψm), ATP and malondialdehyde (MDA), as well as acrosomal integrity, and blastocyst rate and blastocyst quality following IVF. The results contribute to the development of an efficient method for improving the fertilisation capacity of sex-sorted bull sperm, and exploration of the mechanisms involved.

## Results

### Effect of Res on ROS levels in sex-sorted sperm from three bulls

**Figure 1:**
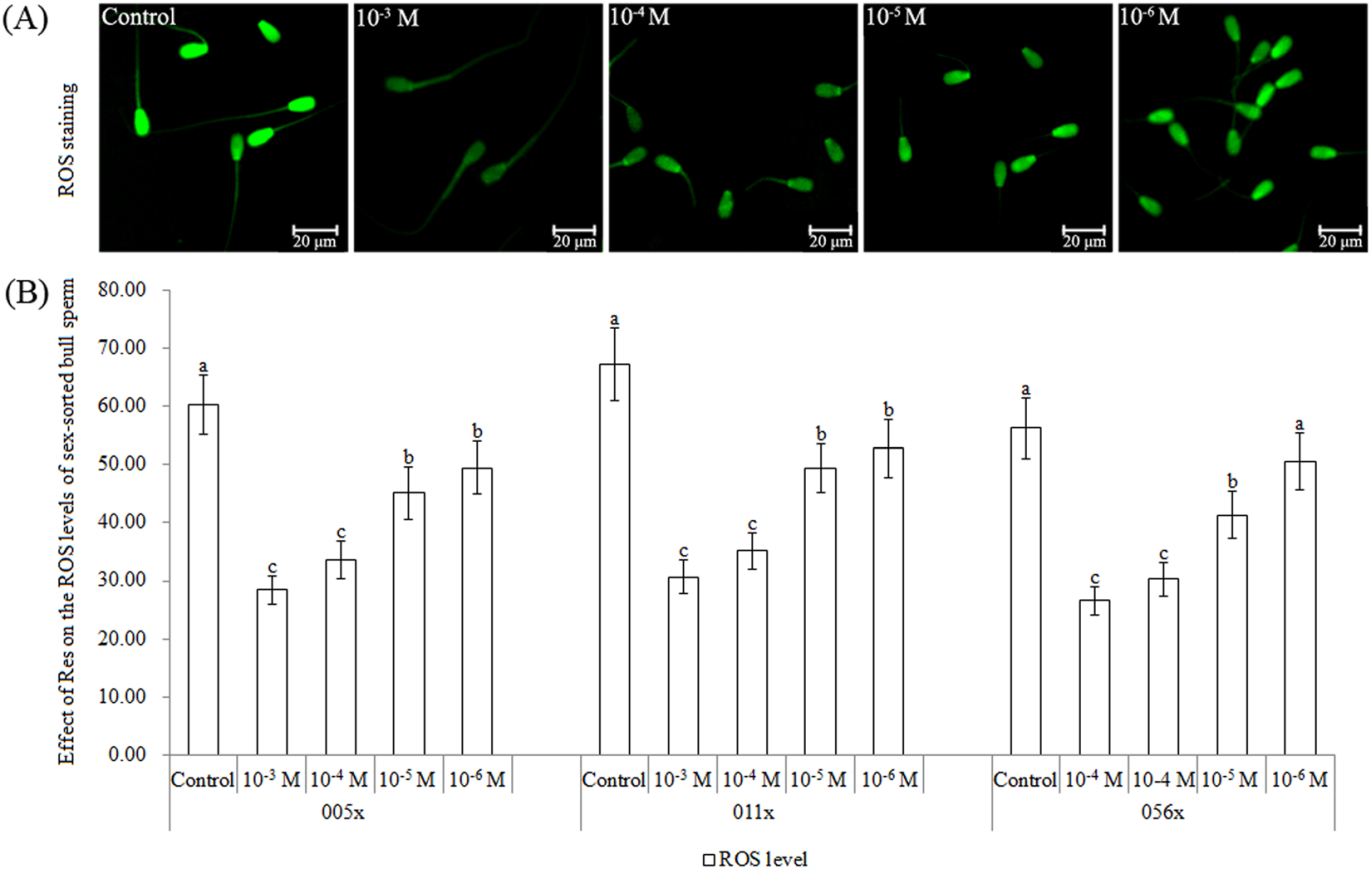
Effect of Res on ROS levels in sex-sorted bull sperm. (**A**) Representative images showing ROS staining. Scale bar = 20 μm. (**B**) ^a,b,c^Values with no common superscript are different (*P* < 0.05).

As shown in Fig. 1B, for bulls 005x, 011x, and 056x, ROS levels in the 10^−4^ M Res group were similar to those in the 10^−3^ M group, but significantly lower than those in the 10^−5^ M group (*P* < 0.05), while ROS levels in these three groups were significantly lower than those in the control group (*P* < 0.05).

### Effect of Res on PS externalisation of bull sex-sorted sperm

**Figure 2:**
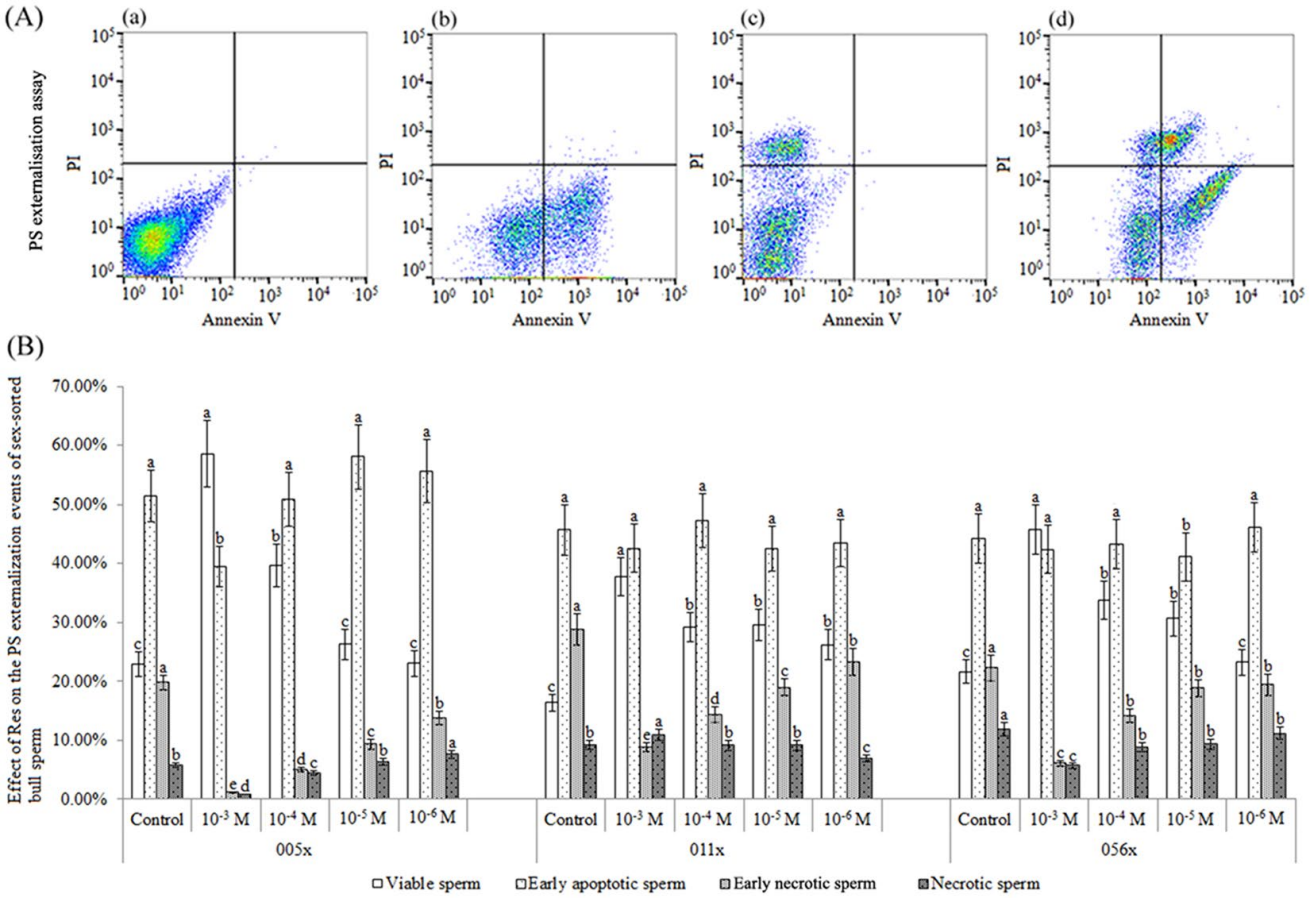
Effect of Res on PS externalisation in sex-sorted bull sperm. (**A**) PS externalisation assay. (a) Negative control. (b) Annexin V FITC staining control. (c) PI staining control. (d) Analysis of sex-sorted bull sperm. Quadrants represent viable sperm (lower-left quadrant), necrotic sperm (upper-left quadrant), early apoptotic sperm (lower-right quadrant), and early necrotic sperm (upper-right quadrant). (**B**) ^a,b,c,d,e^Values with no common superscript are different (*P* < 0.05).

As shown in Fig. 2B, for bulls 005x, 011x, and 056x, the percentage of viable sperm in the 10^−4^ M Res group (39.69 ± 3.64%, 29.17 ± 2.49%, and 33.71 ± 3.25%, respectively) was significantly lower than in the 10^−3^ M Res group (58.58 ± 5.62%, 37.80 ± 3.25%, and 45.82 ± 4.16%; *P* < 0.05), but significantly higher than in controls (22.87 ± 2.15%, 16.38 ± 1.41%, and 21.60 ± 2.01%; *P* < 0.05).

Meanwhile, the percentage of early necrotic sperm in the 10^−3^ M Res group (1.16 ± 0.13%, 8.80 ± 0.81%, and 6.12 ± 0.51%) and the 10^−4^ M Res group (4.97 ± 0.38%, 14.35 ± 1.42%, and 14.20 ± 1.13%) was significantly lower than that of the control group (19.83 ± 1.27%, 28.81 ± 2.74%, and 22.28 ± 2.14%; *P* < 0.05), but no significant differences were found between the percentage of early apoptotic sperm in the control groups (44.25 ± 4.16% to 51.49 ± 4.35%) and 10^−4^ M Res groups (43.31 ± 4.25% to 50.88 ± 4.52%; *P* > 0.05).

### Effect of Res on Δψm in sex-sorted bull sperm membranes

**Figure 3:**
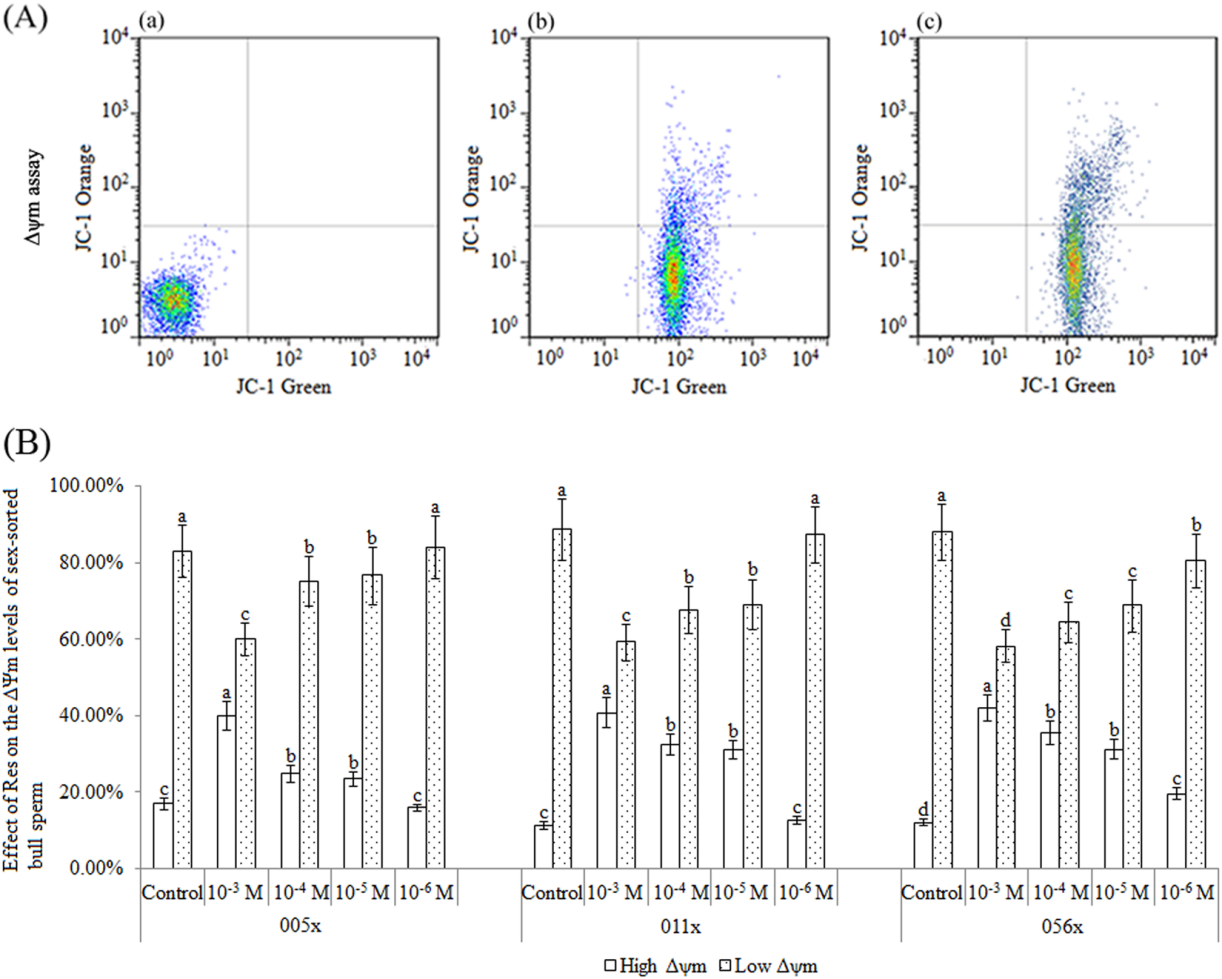
Effect of Res on Δψm in sex-sorted bull sperm membranes. (**A**) Δψm assays. (a) Negative control. (b) Positive control. (c) Analysis of sex-sorted bull sperm. Quadrants represent sperm with low Δψm (lower-right quadrant) and high Δψm (upper-right quadrant). (**B**) ^a,b,c,d^Values with no common superscript are different (*P* < 0.05).

As shown in Fig. 3B, for sex-sorted sperm from bull 005x, the percentage of sperm with a high Δψm value in 10^−3^ M (40.01 ± 3.82%), 10^−4^ M (24.80 ± 2.17%), and 10^−5^ M (23.40 ± 1.83%) Res groups was significantly higher than that of the control group (16.91 ± 1.45%; *P* < 0.05). The 10^−3^ M Res group included the highest percentage of sperm with a high Δψm value, and the same results were found for bulls 011x (40.81 ± 3.92%, 32.43 ± 2.81%, and 31.07 ± 2.24% vs. 11.37 ± 1.06%; *P* < 0.05) and 056x (41.88 ± 3.42%, 35.59 ± 3.06%, and 31.22 ± 2.57% vs. 12.04 ± 0.48%; *P* < 0.05).

Meanwhile, for sex-sorted sperm of bull 005x, the percentage of sperm with a low Δψm value in 10^−3^ M (59.99 ± 4.37%), 10^−4^ M (75.20 ± 6.54%), and 10^−5^ M (76.60 ± 7.48%) Res groups was significantly lower than that of the control group (83.06 ± 6.82%; *P* < 0.05). The 10^−3^ M Res group included the lowest percentage of sperm with a low Δψm value, and the same results were found for bulls 011x (59.18 ± 4.82%, 67.57 ± 6.15%, and 68.94 ± 6.46% vs. 88.63 ± 8.13%; *P* < 0.05) and 056x (58.12 ± 4.27%, 64.41 ± 5.41%, and 68.77 ± 6.75% vs. 87.93 ± 7.32%; *P* < 0.05).

### Effect of Res on ATP content in sex-sorted bull sperm

**Figure 4:**
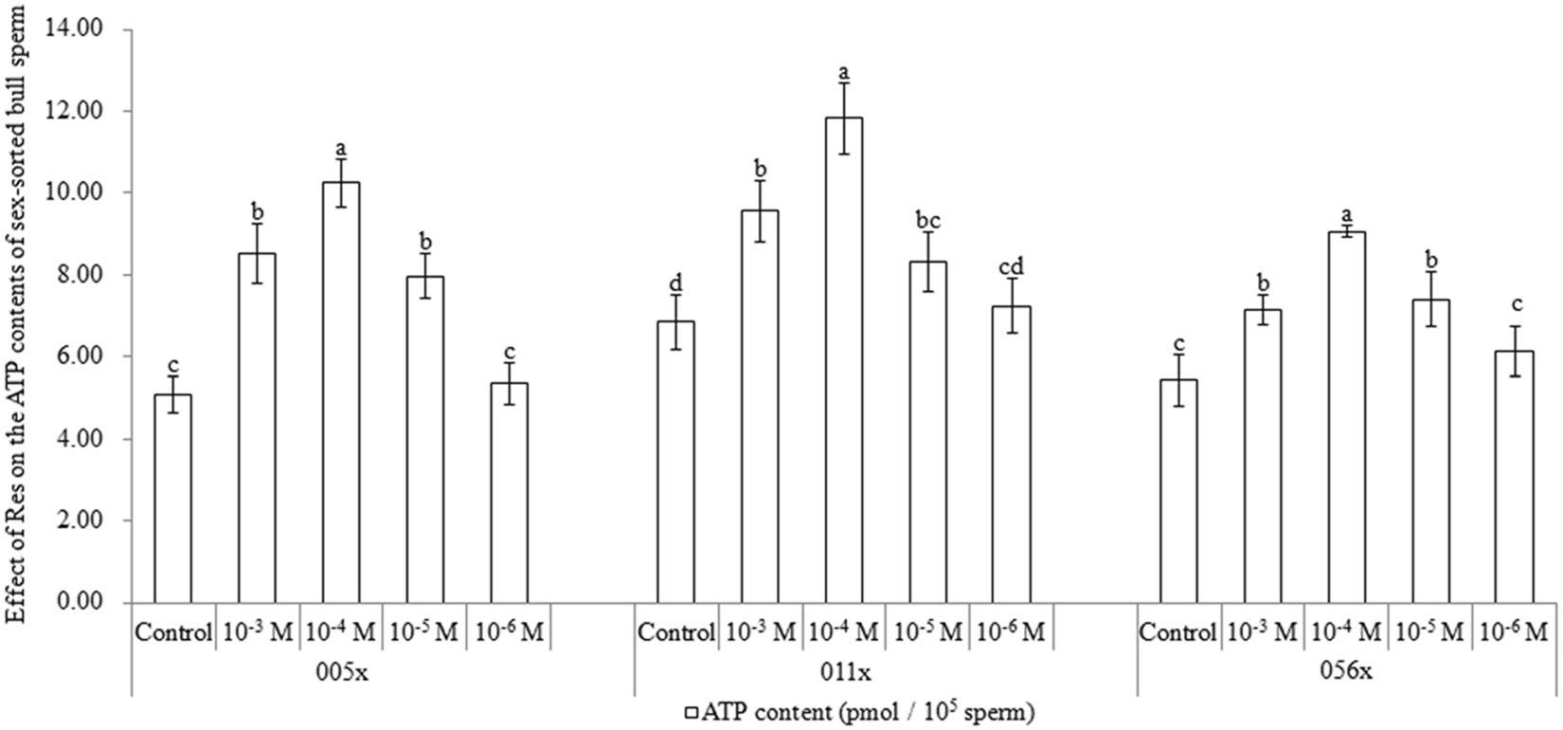
Effect of Res on ATP content in sex-sorted bull sperm. ^a,b,c,d^Values with no common superscript are different (*P* < 0.05).

As shown in Fig. 4, for sex-sorted sperm from bulls 005x, 011x, and 056x, the ATP content in 10^5^ sperm from the 10^−3^ M Res group (8.51 ± 0.73 pmol, 9.56 ± 0.74 pmol, and 7.15 ± 0.35 pmol) was similar to that in the 10^−5^ M Res group (7.97 ± 0.56 pmol, 8.32 ± 0.74 pmol, and 7.40 ± 0.70 pmol), significantly lower than the 10^−4^ M Res group (10.24 ± 0.58 pmol, 11.84 ± 0.87 pmol, and 9.07 ± 0.14 pmol), and significantly higher than controls (5.10 ± 0.44 pmol, 6.85 ± 0.66 pmol, and 5.43 ± 0.64 pmol; *P* < 0.05).

### Effect of Res on MDA content in sex-sorted bull sperm

**Figure 5:**
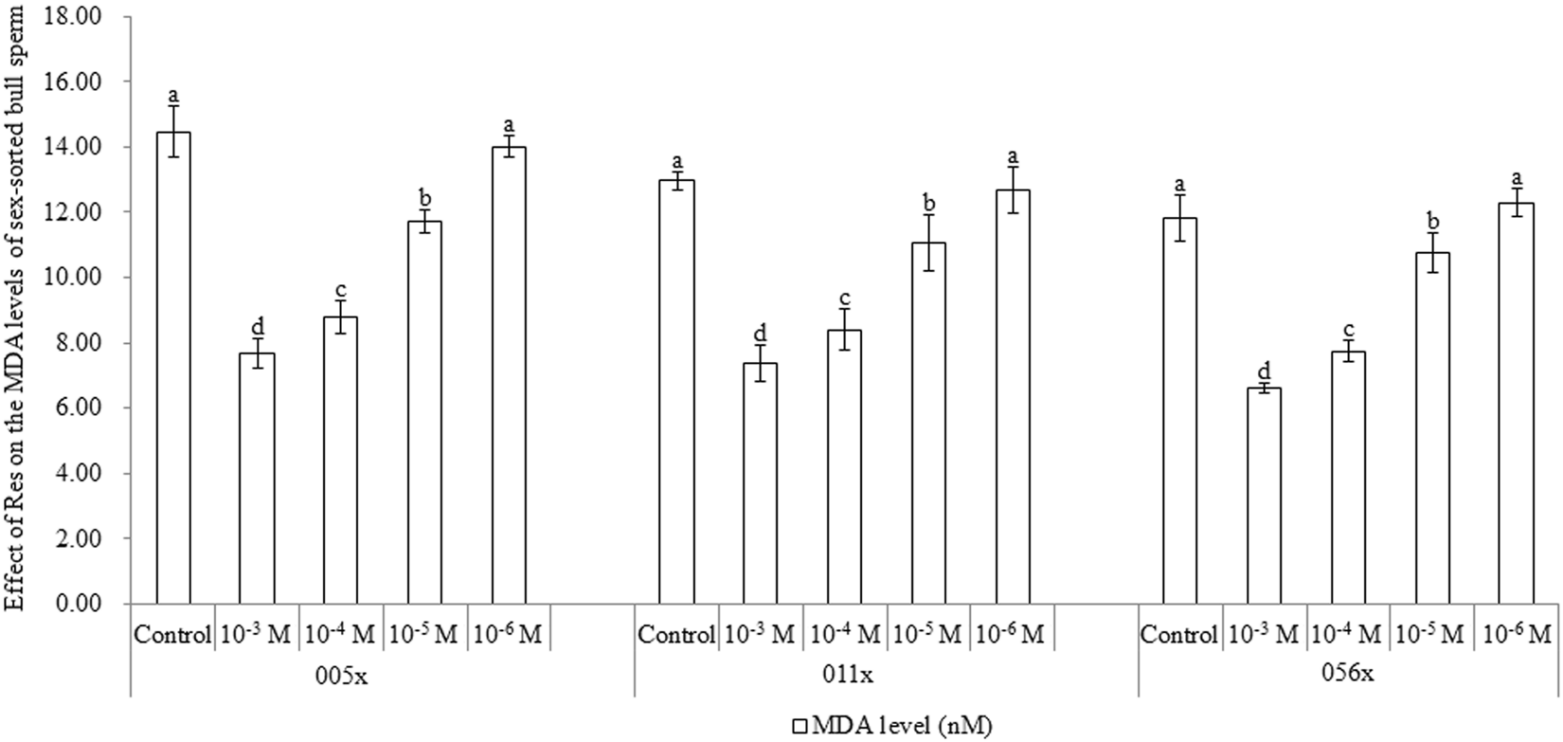
Effect of Res on MDA content in sex-sorted bull sperm. ^a,b,c,d^Values with no common superscript are different (*P* < 0.05).

As shown in Fig. 5, for sex-sorted sperm from bulls 005x, 011x, and 056x, the MDA content in the 10^−3^ M Res group (7.67 ± 0.47 nM, 7.37 ± 0.54 nM, and 6.62 ± 0.14 nM) was significantly lower than that in the 10^−4^ M Res group (8.79 ± 0.49 nM, 8.41 ± 0.65 nM, and 7.75 ± 0.31 nM; *P* < 0.05), and they were all significantly lower than controls (14.47 ± 0.79 nM, 12.96 ± 0.29 nM, and 11.83 ± 0.71 nM; *P* < 0.05).

### Effect of Res on acrosome integrity in sex-sorted bull sperm

**Figure 6:**
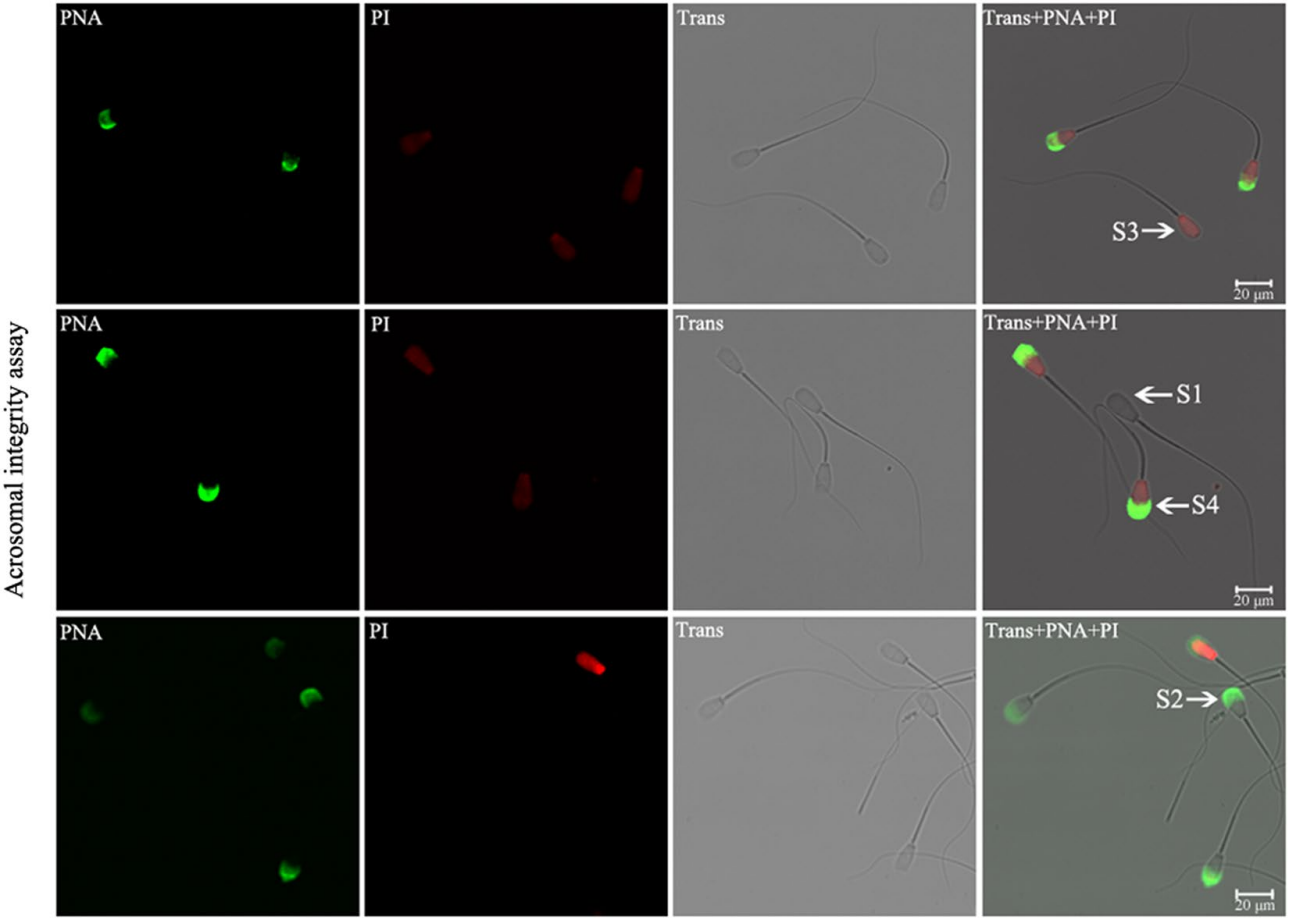
Representative images of acrosome staining of sex-sorted bull sperm. S1, viable sperm with integral acrosomes (PI^−^/PNA^−^). S2, viable sperm with damaged acrosomes (PI^−^/PNA^+^). S3, dead sperm with acrosomal integrity (PI^+^/PNA^−^). S4, dead sperm with damaged acrosomes (PI^+^/PNA^+^). Scale bar = 20 μm.

**Figure 7:**
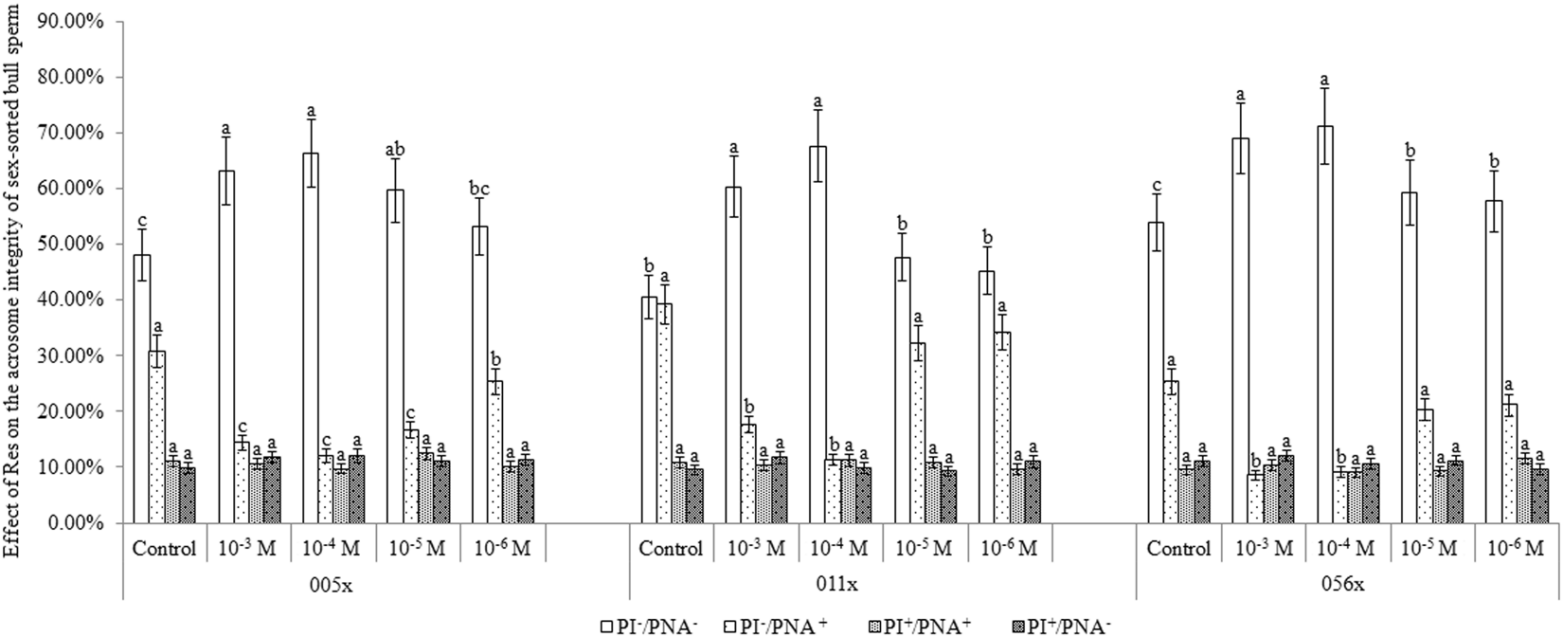
Effect of Res on acrosome integrity in sex-sorted bull sperm. ^a,b,c^Values with no common superscript are different (*P* < 0.05).

Figure 6 shows representative acrosome staining images. As shown in Fig. 7, for sex-sorted sperm from bulls 005x, 011x, and 056x, the percentage of viable sperm with acrosomal integrity in the 10^−4^ M Res group (66.27% ± 6.08%, 67.61 ± 6.38%, and 71.21 ± 6.81%) was similar to the 10^−3^ M Res group (63.16 ± 6.14%, 60.29 ± 5.46%, and 68.97 ± 6.38%), and significantly higher than the control group (48.15 ± 4.62%, 40.54 ± 3.85%, and 53.97 ± 5.06%; *P* < 0.05). Meanwhile, the percentage of viable sperm without acrosomal integrity in the 10^−4^ M Res group (12.05 ± 1.16%, 11.27 ± 1.03%, and 9.09 ± 0.89%) was similar to the 10^−3^ M Res group (14.47 ± 1.32%, 17.65 ± 1.43%, and 8.62 ± 0.82%), and significantly lower than the control group (30.86 ± 2.93%, 39.19 ± 3.61%, and 25.40 ± 2.31%; *P* < 0.05). No significant differences were found in the percentage of dead sperm with or without acrosomal integrity among all five groups for each bull.

### Effect of Res on IVF efficiency in sex-sorted bull sperm

**Figure 8:**
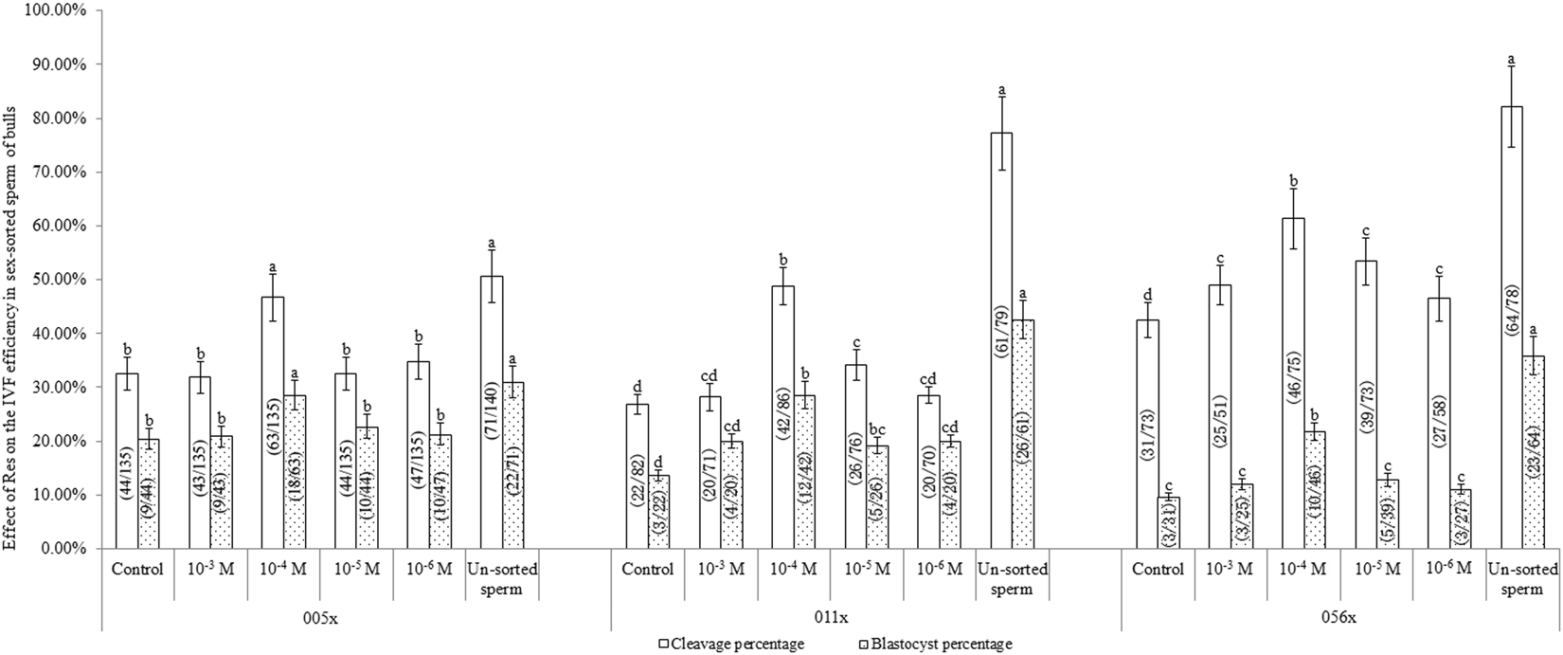
Effect of Res on IVF efficiency in sex-sorted bull sperm. ^a,b,c,d^Values with no common superscript are different (*P* < 0.05).

As shown in Fig. 8, for sex-sorted sperm from bull 005x, the cleavage and blastocyst percentages in the 10^−4^ M Res group (46.67 ± 4.41%, and 28.57 ± 2.68%) were similar to those in un-sorted sperm (50.71 ± 4.89%, and 30.99 ± 2.98%), but significantly higher than the control group (32.59 ± 3.02%, and 20.45 ± 2.01%; *P* < 0.05).

For sex-sorted sperm from bull 056x, cleavage and blastocyst percentages of the 10^−4^ M Res group (61.33 ± 5.65%, and 21.74 ± 1.65%) were significantly lower than those of un-sorted sperm (82.05 ± 7.52%, and 35.94 ± 3.52%; *P* < 0.05), but significantly higher than the control group (42.47 ± 3.26%, and 9.68 ± 0.74%; *P* < 0.05). The same results were found in the cleavage (48.84 ± 3.49% vs. 77.22 ± 6.82%, and 26.83 ± 1.85%; *P* < 0.05) and blastocyst (28.57 ± 2.46% vs. 42.62 ± 3.57%, and 13.64 ± 0.96%; *P* < 0.05) percentages for sex-sorted sperm from bull 011x.

### Effect of Res on the expression of *BAX* and *BCL2L1* mRNAs in bovine blastocysts of sex-sorted bull sperm

**Figure 9:**
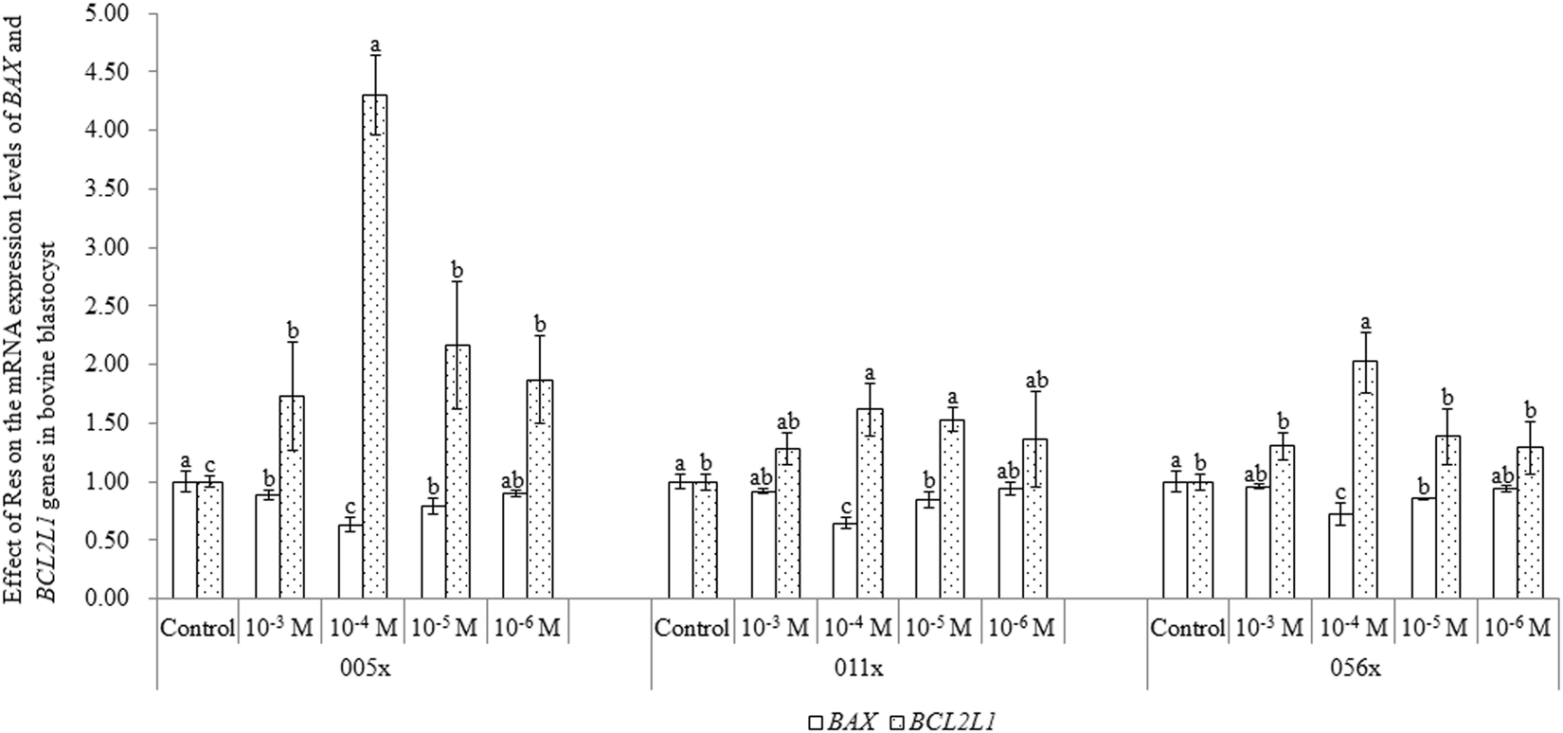
Effect of Res on the expression of *BAX* and *BCL2L1* mRNAs in bovine blastocysts from sex-sorted bull sperm. ^a,b^Values with no common superscript are different (*P* < 0.05).

As shown in Fig. 9, for sex-sorted sperm from bulls 005x, 056x, and 011x, expression levels of *BAX* mRNA in the 10^−4^ M and 10^−5^ M Res groups were significantly lower than controls (*P* < 0.05). By contrast, *BCL2L1* mRNA expression levels in the 10^−4^ M Res groups were significantly higher than controls (*P* < 0.05).

## Discussion

### Effect of Res on ROS levels in sex-sorted bull sperm

It has been reported that Res supplementation in the extender before cryopreservation significantly decreases oxidative stress in buffalo sperm^17^, and Res treatment significantly decreased ROS levels in mouse sperm^11^. As shown in Fig. 1, Res supplementation in washing and fertilisation medium significantly decreased ROS levels in sex-sorted bull sperm. Res is a powerful antioxidant due to its ability to inhibit ROS formation by enzymatic and non-enzymatic systems, and its ROS scavenging activity^14^, which helps to explain these results.

### Effect of Res on PS externalisation in sex-sorted bull sperm

PS externalisation is considered an early apoptotic marker. As shown in Fig. 2, 10^−3^ M or 10^−4^ M Res treatment significantly increased the percentage of viable sperm. It has been reported that oxidative stress leads to PS translocation in human sperm^18,19^, which helps to explain the improved percentage of viable sperm in 10^−3^ M and 10^−4^ M Res groups containing lower ROS levels (Fig. 1).

### Effect of Res on Δψm values in sex-sorted bull sperm

Mitochondria play a significant role in the apoptotic process^20^, and impaired mitochondrial function results in diminished fertility and motility in sperm from human^21^ and ram^22^. The Δψm parameter is an important indicator of mitochondrial function, and its measurement provides useful information on the ability of sperm to fertilise eggs^23^. Oxidative stress can lower the Δψm value in human sperm^24,25^, and loss of Δψm decreases the ability of sperm to trigger the acrosome reaction^26^, impairs ATP synthesis, and increases ROS generation^27^.

It has been reported that supplementation of bull semen extender with Res increases mitochondrial activity^12^. As shown in Fig. 3, our results also showed that Res treatment (10^−3^ M, 10^−4^ M, and 10^−5^ M) significantly increased Δψm in sex-sorted bull sperm, presumably due to lower ROS levels in these groups (Fig. 1) because oxidative stress is known to decrease Δψm in human sperm^24,25^.

### Effect of Res on ATP content in sex-sorted bull sperm

An increase in ROS content can target mitochondria and affect ATP production^28^, and the Δψm value is positively correlated with the ATP content in sperm^29^. This may help to explain the higher ATP content in the 10^−3^ M, 10^−4^ M and 10^−5^ M Res groups (Fig. 4), given their lower ROS content and higher Δψm values.

### Effect of Res on MDA content in sex-sorted bull sperm

Mammalian spermatozoa, especially frozen sperm, are highly sensitive to LPO^6^, which is formed following oxidation of membrane lipids by ROS^30^. MDA is the main byproduct of LPO, which aside from its longevity, can easily diffuse across membranes and react with proteins or DNA to form adducts^31^. Similar to previous reports^20,32^, our current results also showed that 10^−3^ M or 10^−4^ M Res treatment significantly decreased the MDA content in sex-sorted bull sperm (Fig. 5), presumably due to their lower ROS levels, as described above.

### Effect of Res on acrosome integrity in sex-sorted bull sperm

ROS impairs the acrosome membrane of sperm^33^. Moreover, polyunsaturated fatty acids (PUFAs) located in sperm are sensitive to the LPO cascade, resulting in sperm membrane lipid peroxidation injury and changes to the liquidity and integrity of the membrane, which can result in acrosome breakage and leakage of the contents, culminating in sperm-egg recognition errors^34^. Moreover, it has been proven that Res plays an important role in preventing premature sperm capacitation and, consequently, acrosome reaction^35,36^. Together, these results can help explain the higher percentage of sex-sorted sperm in which the integrity of the acrosome is preserved in the 10^−3^ M or 10^−4^ M Res groups, as shown in Fig. 7.

### Effect of Res on IVF efficiency in sex-sorted bull sperm

It has been reported that 50 μM Res supplementation in the cryopreservation extender significantly increases the normal fertilisation rate of buffalo oocytes^17^, and 2 mM Res supplementation in thawing extender exerts a positive effect on the total efficiency of fertilisation in boar sperm^13^. Similarly, our experiments showed that 10^−4^ M Res supplementation in washing and fertilisation medium of sex-sorted bull sperm significantly increased the cleavage and blastocyst percentage in oocytes following IVF (Fig. 8).

ROS can induce LPO and DNA fragmentation in sperm, decreasing sperm motility^37^ and lowering the Δψm value in human sperm^24,25^, and diminishing the ability of sperm to trigger the acrosome reaction^26^. Res can protect bull sperm motility^12^ and prevent premature capacitation of buffalo sperm by protecting membranes from toxic oxygen metabolites^17^. In our present study, Res significantly decreased ROS levels (Fig. 1), PS externalisation (Fig. 2), and MDA content (Fig. 5), and increased the Δψm value (Fig. 3) and ATP content (Fig. 4), which helps to explain the increased cleavage and blastocyst percentages of sex-sorted bull sperm following IVF (Fig. 8).

### Effect of Res on the expression of *BAX* and *BCL2L1* mRNAs in bovine blastocysts of sex-sorted bull sperm

The BAX protein enhances the release of cytochrome c from mitochondria and thereby promotes apoptosis^38^, while the BCL2L1 protein inhibits the release of cytochrome c from mitochondria, preventing apoptosis^39^. In our experiments, Res treatment significantly increased the expression of *BCL2L1* mRNAs and decreased the expression of *BAX* mRNAs (Fig. 9), indicating that Res treatment of sex-sorted sperm inhibits the apoptotic pathway in blastocysts obtained following IVF.

In conclusion, our results showed that treatment with 10^−4^ M Res significantly decreased the levels of ROS, MDA, and PS externalisation, and protected mitochondrial function and acrosomal integrity in sex-sorted bull sperm, thereby increasing their blastocyst percentage and blastocyst quality following IVF.

## Materials and Methods

### Chemicals and Ethics statement

Unless otherwise specified, all chemicals and reagents used in the experiments were purchased from Sigma Chemical Co. (St. Louis, MO, USA). All treatment of animals was performed according to the requirements of the Institutional Animal Care and Use Committee of the Chinese Academy of Agricultural Sciences. Before the start of this experiment, the protocol was approved by the same committee, including feeding, management of animals, and so on.

### Oocyte recovery and *in vitro* maturation (IVM)

Bovine ovaries were transported from a local slaughterhouse to the laboratory within 2 h of collection. Cumulus-oocyte complexes (COCs) were aspirated from follicles (2−8 mm in diameter), and only those surrounded with at least three layers of cumulus cells were selected for IVM. IVM medium contained HEPES-buffered TCM-199 (Earle’s salt; Gibco BRL, Grand Island, NY, USA) enriched with 10 μg/mL follicle stimulating hormone (FSH), 10% (v/v) fetal bovine serum (FBS), 10 μg/mL luteinising hormone (LH), 10 μg/mL heparin, and 1 μg/mL estradiol. For IVM, groups of 50 COCs were incubated in four-well plates containing 500 μL IVM-culture drops under mineral oil and incubated in a humidified atmosphere at 38.5 °C in 5% CO_2_ for 22–24 h.

### Preparation of sex-sorted semen

Frozen sex-sorted bovine sperm were purchased from OX Livestock Breeding Co. Ltd (Shandong, China). During the thawing procedure, frozen sex-sorted semen was removed from liquid nitrogen and placed in a water bath at 38 °C for 1 min, then transferred to 15 mL sterile conical centrifuge tubes and mixed with 3 mL pre-heated washing medium (Brackett and Oliphant medium supplemented with 2.5 mM caffeine and different concentrations of Res). Subsequently, they were washed by centrifuging for 5 min at 328 × g. After centrifugation, the sperm pellet was suspended in fertilisation medium (Brackett and Oliphant medium contain 20 mg/mL BSA, 20 μg/mL heparin, 100 IU/mL penicillin, 100 μg/mL streptomycin, and different concentrations of Res) to a final concentration of 1 × 10^6^/mL. Resuspended sperm were then incubated at 38.5 °C in 5% CO_2_ for 1.5 h before use in subsequent experiments.

### Analysis of ROS levels

According to the method described by Barroso *et al*.^40^, 2′,7′-dichlorodihydrofluorescein diacetate (H2DCFDA) was used to analyse ROS levels in sperm. Briefly, each sperm group was incubated in 10 μM H2DCFDA for 20 min at 38.5 °C in the dark. Samples were then washed and centrifuged for 5 min at 328 × g, resuspended in phosphate-buffered saline (PBS), and analysed by a laser confocal microscope (Leica, SP8, Germany) to measure ROS levels within cells.

### Analysis of PS externalisation events

An Annexin V-FITC kit (Biovision, Mountain View, CA, USA) was used to detect PS externalisation events in sperm. In brief, a sperm pellet prepared as described above was supplemented with 1 mL of 1× Annexin V binding buffer, then washed by centrifuging for 5 min at 328 × g. Subsequently, the sperm pellet was incubated in 5 μL Annexin V-FITC and 5 μL propidium iodide (PI) at 38.5 °C for 5 min in the dark. Samples were analyzed by flow cytometry (MoFlo XDP, Beckman, USA), and a total of 10,000 sperm were analysed for each group. Sperm stained and unstained with Annexin V-FITC or PI were used as controls.

According to the method described by Anzar *et al*.^41^, sperm were separated into four groups based on staining results; viable sperm (Annexin V-FITC^−^ and PI^−^), early apoptotic sperm (Annexin V-FITC^+^ and PI^−^), early necrotic sperm (Annexin V-FITC^+^ and PI^+^), and necrotic sperm (Annexin V-FITC^−^ and PI^+^). Each experiment was repeated at least three times.

### Analysis of Δψm using a JC-1 probe

A JC-1 probe (MitoProbe JC-1 assay kit; Invitrogen) was used to evaluate the Δψm value of sex-sorted bull sperm according to the method described previously^42^. Each sperm group was incubated in 2 μM JC-1 for 30 min at 38.5 °C in the dark, and samples were washed then analysed using flow cytometry. A total of 10,000 sperm were analysed for each group. Meanwhile, unstained sperm were used as negative controls, and sperm exposed to ultraviolet light for 30 min were used as positive controls.

### Analysis of ATP content

A bioluminescence assay kit (ATP Bioluminescence Assay Kit HS II, Roche Diagnostics GmbH, Mannheim, Germany) was used to measure the ATP content of sex-sorted sperm. Briefly, sperm from each group were added to extraction medium consisting of 100 mM TRIS-HCl (pH 7.75) and 4 mM EDTA. The sperm suspension was boiled for at 100 °C for 2 min, then centrifuged at 12,000 × g for 20 min. The ATP content of the supernatant was measured using a Bioluminescence Assay Kit. The luminescence was read using a luminometer (InfniteM200, Tecan Group Ltd., Untersbergstrasse, Austria), and data expressed as picomoles (pmol) of ATP per 10^5^ sperm.

### Analysis of MDA content

An MDA kit (Nanjing Jiancheng Bioengineering Institute, China) was used to measure the MDA level in sperm samples. For this assay, each sperm group was incubated for 80 min in 0.5% TBA, followed by rapid cooling and acquisition of the supernatant by high-speed centrifugation, and samples were injected directly into a spectrophotometer (Beckman Coulter, Inc., CA, USA). MDA levels are expressed in nM units.

### Analysis of acrosomal integrity by FITC-PNA

According to the method described previously^43^, sperm were stained with 5 µg/mL FITC-PNA and 50 µg/mL PI and incubated at 38.5 °C in the dark for 10 min. Sperm were then washed and observed by confocal microscopy (Nikon, Tokyo, Japan). The percentage of viable sperm with integral acrosomes (PI^−^/PNA^−^), viable sperm with damaged acrosomes (PI^−^/PNA^+^), dead sperm with acrosomal integrity (PI^+^/PNA^−^), and dead sperm with damaged acrosomes (PI^+^/PNA^+^) was then determined.

### IVF experiments

After IVM, COCs were transferred into 0.1% (w/v) hyaluronidase for 30 s and gently pipetted to remove some of the cumulus cells. Only oocytes with the first poly body and homogenous cytoplasm were selected for IVF. Sex-sorted sperm were washed and diluted to achieve a concentration of 5 × 10^6^/mL as mentioned above. A 20 μL aliquot of this suspension was added to 80 μL droplets of fertilisation medium containing 20–30 oocytes under mineral oil, and incubated at 38.5 °C in air with 5% CO_2_ for insemination. After 16−18 h, presumptive zygotes were transferred and cultured in CR1aa culture medium (Rosenkrans and First, 1994) for 48 h. Cleaved embryos were then recorded and cultured in CR1aa medium containing 10% FBS for 5 days. The medium was changed every 2 days throughout the culture period. To evaluate the effect of Res on fertilisation, cleavage and blastocyst percentages were recorded on days 2 and 7 after fertilisation.

### Analysis mRNA levels of apoptotic genes in blastocysts following IVF

A single Cell-to-CT quantitative real-time PCR kit (Life Technologies, Carlsbad, CA, USA) and the 7900HT system (Applied Biosystems, Foster City, CA, USA) were utilised to measure mRNA levels of apoptotic genes in blastocysts as described previously^44^. The PCR procedure was carried out in 10 μL mixtures containing 2 μL complementary DNA, 5 μL Fast SYBR Green Master Mix (Invitrogen), and 0.2 μL of each primer (10 μM). Primers are listed in Table 1^44^. The results were analysed by the comparative Ct (2^−ΔΔCt^) method with *Β-ACTIN* as the reference gene.

**Table 1:** Sequences of primers used to amplify the selected blastocyst genes^44^.

Gene	Primers (5′-3′)	GeneBank accession No.
*BAX*	F: TTCTGACGGCAACTTCAACTG	NM_173894.1
	R: CTCTCGAAGGAAGTCCAATGTC	
*BCL2L1*	F: AGGAGATGCAGGTATTGGTGA	NM_001077486.2
	R: CATTGTTCCCGTAGAGTTCCA	
	R: CATCTCCTGAGGAAGACCAAA	
*Β-ACTIN*	F: GGGAAATCGTCCGTGACATCA	NM_173979
	R: GATGGTGATGACCTGCCCGT	

### Experimental design

To investigate the effect of Res on the quality of sex-sorted bull semen and the mechanisms involved, five different concentrations of Res (0, 10^−3^, 10^−4^, 10^−5^, and 10^−6^ M) were supplemented in the washing medium and fertilisation medium for bulls 005x, 011x, and 056x, and levels of ROS, PS externalisation, Δψm, ATP, MDA, and acrosomal integrity, blastocyst rate, and quality following IVF were determined.

### Statistical analysis

Data are expressed as mean ± standard error. All biological experiments were repeated at least three times. All data were analysed using Statistical Analysis System software (SAS Institute Inc., Cary, NC, USA) to perform one-way analysis of variance (ANOVA) using Duncan’s tests. For all analyses, the results were considered statistically significant at *P* < 0.05.
